# Survival among women diagnosed with screen-detected or interval breast cancer classified as true, minimal signs, or missed through an informed radiological review

**DOI:** 10.1007/s00330-020-07340-4

**Published:** 2020-11-12

**Authors:** Kaitlyn M. Tsuruda, Tone Hovda, Sameer Bhargava, Marit B. Veierød, Solveig Hofvind

**Affiliations:** 1 Section for Breast Cancer Screening, Cancer Registry of Norway, PO Box 5313, Majorstuen, 0304 Oslo, Norway; 2 Oslo Centre for Biostatistics and Epidemiology, Department of Biostatistics, Institute of Basic Medical Sciences, University of Oslo, PO Box 1122, Blindern, 0317 Oslo, Norway; 3 Department of Radiology, Vestre Viken Hospital Trust, PO Box 800, 3004 Drammen, Norway; 4 Institute of Clinical Medicine, University of Oslo, PO Box 1171, Blindern, 0318 Oslo, Norway; 5 Division of Oncology, Department of Medicine, Bærum Hospital, Vestre Viken Hospital Trust, PO Box 800, 3004 Drammen, Norway; 6 Faculty of Health Sciences, Oslo Metropolitan University, Pilestredet Campus, PO Box 4 St. Olavs plass, N-0130 Oslo, Norway

**Keywords:** Breast neoplasms, Mammography, Mass screening, Survival rate, Early detection of cancer

## Abstract

**Objectives:**

“True” breast cancers, defined as not being visible on prior screening mammograms, are expected to be more aggressive than “missed” cancers, which are visible in retrospect. However, the evidence to support this hypothesis is limited. We compared the risk of death from any cause for women with true, minimal signs, and missed invasive screen-detected (SDC) and interval breast cancers (IC).

**Methods:**

This nation-wide study included 1022 SDC and 788 IC diagnosed through BreastScreen Norway during 2005–2016. Cancers were classified as true, minimal signs, or missed by five breast radiologists in a consensus-based informed review of prior screening and diagnostic images. We used multivariable Cox regression to estimate hazard ratios (HRs) and 95% confidence intervals (CIs) for the risk of death from any cause associated with true, minimal signs, and missed breast cancers, adjusting for age at diagnosis, histopathologic tumour diameter and grade, and subtype. Separate models were created for SDC and IC.

**Results:**

Among SDC, 463 (44%) were classified as true and 242 (23%) as missed; among IC, 325 (39%) were classified as true and 235 (32%) missed. Missed SDC were associated with a similar risk of death as true SDC (HR = 1.20, 95% CI (0.49, 2.46)). Similar results were observed for missed versus true IC (HR = 1.31, 95% CI (0.77, 2.23)).

**Conclusions:**

We did not observe a statistical difference in the risk of death for women diagnosed with true or missed SDC or IC; however, the number of cases reviewed and follow-up time limited the precision of our estimates.

**Key Points:**

*• An informed radiological review classified screen-detected and interval cancers as true, minimal signs, or missed based on prior screening and diagnostic mammograms.*

*• It has been hypothesised that true cancers, not visible on the prior screening examination, may be more aggressive than missed cancers.*

*• We did not observe a statistical difference in the risk of death from any cause for women with missed versus true screen-detected or interval breast cancers.*

**Supplementary Information:**

The online version of this article (10.1007/s00330-020-07340-4) contains supplementary material, which is available to authorized users.

## Introduction

Mammographic screening is considered the best approach to detect breast cancer at an early stage and thereby reduce breast cancer mortality [1, 2]. For mass screening to be effective, radiologist sensitivity, the ability to correctly identify women *with* breast cancer, must be balanced against specificity, the ability to correctly identify women *without* breast cancer. False-positive screening exams are associated with temporary uncertainty and anxiety [3–5], and healthcare costs for further assessment [6]; however, this follow-up can provide confirmation that a woman does not have breast cancer. On the other hand, false negatives may lead to a delayed diagnosis of breast cancer and can lower women’s confidence in mammographic screening [7].

Retrospective radiologic reviews of prior screening mammograms can give insights into the effectiveness and quality of mammographic screening [1, 8]. These reviews are typically limited to the prior screening mammograms of interval breast cancers, but can also include the prior screening mammograms of screen-detected breast cancers and are often performed with access to diagnostic mammograms [9–11]. Typically, reviewing radiologists classify cancers as “true”, “minimal signs”, “missed”, or “occult” [1]. Cancers that are classified as not visible on prior screening mammograms but that develop following a true-negative screening examination are considered true. Minor abnormalities that are regarded as visible on the prior screening mammograms but did not lead to a diagnosis of breast cancer are considered minimal signs. Cancers that are retrospectively visible on prior screening mammograms are considered missed at the prior screen and the prior screening examination is then considered a false negative. Occult cancers are those that are not regarded as mammographically visible at diagnosis but may be symptomatic or diagnosed through other modalities such as ultrasound. The rates of these types of cancers in an organised screening program are associated with the sensitivity of the interpreting radiologists and that of any follow-up assessment, as well as the review process [12–15].

The histopathology of true, minimal signs, and missed screen-detected breast cancers is not well described [9, 16]. True interval breast cancers have more often been histopathologic grade 3 with a smaller tumour diameter than missed breast cancers, but other aspects of histopathology have not demonstrated consistent results [17–22]. It has been hypothesised that the short sojourn time of true breast cancers indicates that they are more aggressive than missed breast cancers [9, 23]. However, we are not aware of any studies that report the survival of women with true and missed screen-detected breast cancer, and three of four studies did not observe a difference in the overall survival of women with true and missed interval breast cancers [18, 19, 21, 23]. These survival results are based on decades-old data, and breast cancer screening, diagnosis, and treatment have since improved considerably [8, 24].

Radiologic reviews often aim to understand the distribution of true, minimal signs, and missed cancers and reduce the rate of missed cancers in order to improve the quality of mammographic screening. However, it is also important to evaluate whether having a breast cancer classified as true, minimal signs, or missed has prognostic implications for women attending screening. The objective of this retrospective study was to re-use data from a completed informed radiologic review to describe the histopathologic findings and survival associated with these three classifications of screen-detected and interval breast cancers within a population-based breast cancer screening program.

## Methods

This study was part of a project approved by the Oslo University Hospital data protection official for research (PVO 2016-4696).

### Retrospective radiological review

The radiologic classifications used in this study were obtained during a nationwide, multicentre informed review of ductal carcinoma in situ (DCIS) and invasive breast cancer diagnosed in BreastScreen Norway. The Cancer Registry of Norway (CRN) administers this population-based screening program and also administered this review, which was performed between September 2016 and April 2017. The review included digital mammograms from consecutive round screen-detected breast cancers and interval breast cancers. BreastScreen Norway offered screening with digital mammography in a single-centre study starting in 2000, and this technology was implemented at all 16 centres in the program by the fall of 2011 [25]. Screen-detected cancer was defined as breast cancer diagnosed after a recall for further assessment due to abnormal mammographic findings, and interval cancer was defined as breast cancer diagnosed within 24 months of a negative screen, or 6–24 months after a false-positive screening result.

The review was designed to include a stratified sample of 75 screen-detected and 75 interval cancers diagnosed at each of the 16 breast centres. With respect to the national distribution of breast cancers, this non-proportional sampling method over-represented smaller centres and under-represented larger centres. However, this gave participating radiologists an equal opportunity to review and learn from cases diagnosed within their own centres. Screen-detected cancers were oversampled at low-volume centres with too few interval cancers diagnosed after screening with digital mammography. Recently diagnosed cancer cases were preferred over older cases to facilitate retrieval from the picture archiving and communication systems (PACS). The review is described elsewhere [26].

Briefly, the review was performed at the breast centres by a pool of 37 radiologists who had read at least 5000 mammograms during the past 2 years. The centres were randomly paired and radiologists from paired centres reviewed each other’s cases. A panel of five radiologists reviewed each case: two from the reviewing centre, two from the paired centre, and one (T.H.) was present at every session. The panel had access to screening and diagnostic images. Through consensus, or a majority vote if consensus could not be reached, the panel of radiologists classified all cancer cases as “true”, “minimal signs, actionable”, “minimal signs, non-actionable”, “missed”, or “occult” indicating whether a cancer was visible and/or perceived at the prior screening examination (Table 1). Information on surgical treatment and histopathology was provided after a case was reviewed.

**Table 1:** Definitions of radiological and study classifications of true, minimal signs, and missed screen-detected and interval breast cancers

Radiological classification	Study classification	Definition
True	True	No abnormalities visible on prior screening mammograms at the cancer site (true-negative prior screen), followed by a diagnosis of interval breast cancer, or screen-detected breast cancer during the subsequent screening round
Minimal signs, actionable	Minimal signs	Minor abnormalities visible on prior screening mammograms at the cancer site. Recall would have warranted, but was not expected within the screening program
Minimal signs, non-actionable	Minimal signs	Non-specific findings visible on prior screening mammograms at the cancer site. Recall not possible or expected within the screening program
Missed	Missed	Obvious abnormalities visible on prior screening mammograms at the cancer site (false negative prior screen) that resulted in interval breast cancer or screen-detected breast cancer during the subsequent screening round
Occult	Excluded	No mammographically visible findings at diagnosis

### Study sample

**Fig. 1:**
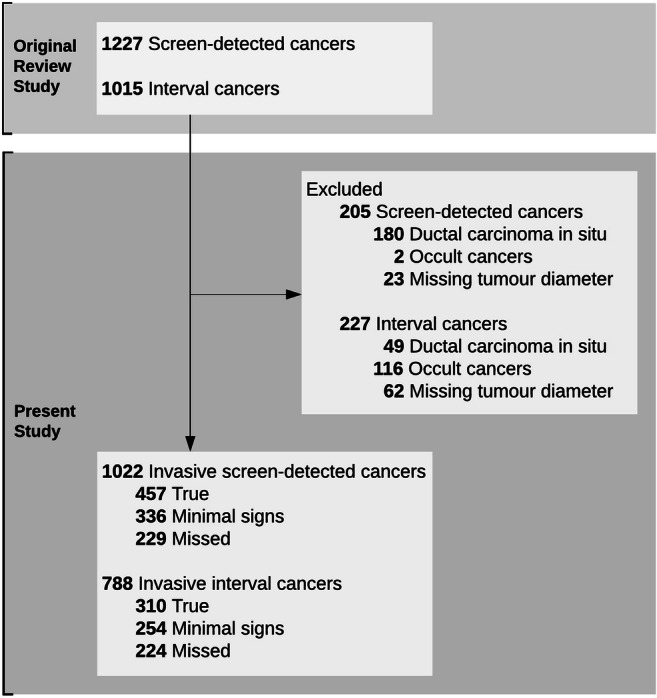
Number of individuals included and excluded. Individuals were excluded sequentially using the exclusion criteria

The review included mammograms from 1227 screen-detected and 1015 interval breast cancers (both DCIS and invasive). However, in this study, we sequentially excluded DCIS, occult cases, and cases for whom information about postoperative histopathological tumour diameter was unavailable (Fig. 1). The proportion of DCIS in the review reflected population averages in BreastScreen Norway [25]. Women with DCIS in Norway are typically treated with breast-conserving surgery and adjuvant radiation therapy, which is associated with low long-term rates of local recurrence [27]. We excluded women with DCIS because the excellent survival in this group makes it difficult to conduct an informative survival analysis. The largest tumour was included for multifocal (*n* = 44 screen-detected and 36 interval) and bilateral cancers (*n* = 21 screen-detected and 15 interval).

### Data sources and variables

Three radiological review classifications were used in this study: true, minimal signs, and missed. The minimal signs classification included both actionable and non-actionable cases (Table 1).

The CRN provided information about cancer diagnoses and prior screening exams, including women’s age at diagnosis, date of screening and diagnosis, screening location, and mode of detection (screen-detected or interval). The CRN also provided information on histopathologic type (invasive no special type (NST), lobular, other), histopathologic tumour diameter (mm), histopathologic grade (1, 2, 3), lymph node status, estrogen receptor (ER) and progesterone receptor (PR) status, Ki67 expression, and human epidermal growth factor receptor 2 (HER2) status. Breast cancer subtype (Luminal A-like, Luminal B-like, HER2+, triple negative) was determined by applying a clinicopathologic surrogate definition of intrinsic subtypes to ER, PR, Ki67, and HER2 information [28]. These variables are described in detail in Table S1.

Women were followed from date of histologically verified invasive breast cancer until death, emigration, or December 31, 2018. Information on death and emigration was obtained from the CRN, which regularly receives information from the national Cause of Death Registry [29].

### Statistical analysis

Descriptive results were presented as proportions (95% confidence intervals, CIs, calculated using the Wilson score interval [30]), means (standard deviations, SDs), and medians (95% CIs from quantile regression with standard errors based on 100 bootstrap replications).

Kaplan-Meier estimates were used for overall survival in true, minimal signs, and missed cancers. Nelson-Aalen cumulative hazard estimates were used to estimate the risk of breast cancer death. Differences between true, minimal signs, and missed cancers were tested using the log-rank test. We used Cox regression with time since diagnosis as the time variable to estimate hazard ratios (HRs) with 95% CIs for the risk of death from all causes in true, minimal signs, and missed cancers. We adjusted for age at diagnosis and included tumour diameter, grade, and subtype as confounders based on a priori knowledge of their relationship with the exposure and outcome in interval cancers [17–22]. All analyses were conducted separately for screen-detected and interval cancers due to the potential for lead time bias in combined analyses. The proportional hazards assumption was checked using graphical methods and Schoenfeld residuals [31]. This assumption was initially violated in the analysis of interval cancers, but was satisfied after splitting the follow-up time after 3 years, and stratifying on subtype.

Multiple imputation with chained equations was used to impute missing data for grade; lymph node status; ER, PR, and HER2 positivity; and Ki67 expression. Subtype was determined after imputation. Given detection mode and year of diagnosis, data were assumed to be approximately missing completely at random. To increase predictive power, the imputation models also included the radiological classification (true, minimal signs, and missed), tumour diameter, screening centre, age at diagnosis, information about whether women were alive at the end of follow-up, and the Nelson-Aalen cumulative hazard estimator for overall survival [32]. We presented results based on 40 imputed data sets.

We conducted a sensitivity analysis in which we included women without tumour diameter information, and did not use tumour diameter as a covariate in the imputation or Cox regression models.

All analyses were performed using Stata version 16.0 (StataCorp).

## Results

The final study sample consisted of 1022 screen-detected and 788 interval cancers with prior screening examinations between January 2005 and March 2016 (Fig. 1). Among screen-detected cancers, 457 (45%) were classified as true, 336 (33%) as minimal signs, and 229 (22%) as missed. Among interval cancers, 310 (39%) were classified as true, 254 (32%) as minimal signs, and 224 (28%) as missed.

Mean (SD) age at diagnosis did not differ by more than 2 years for women with true (62 (5.1)), minimal signs (62 (4.7)), or missed (63 (4.8)) screen-detected cancer, or for women with true (59 (5.8)), minimal signs (60 (5.7)), or missed (61 (5.2)) interval cancer.

### Histopathologic findings

True screen-detected cancers had less favourable histopathology than minimal signs and missed cancers, which had comparable histopathology (Table 2). In particular, true screen-detected cancers had a higher proportion of grade 3 tumours (30.0%) than minimal signs (14.9%), or missed cancers (13.7%), and were more likely to be triple negative (9.8% versus 2.3% and 2.9%). True interval cancers also had less favourable histopathology than minimal signs and missed interval cancers, which generally had comparable histopathologic characteristics. True interval cancers were more likely to be grade 3 (46.7%) than minimal signs (36.1%), or missed cancers (35.9%). The proportion of triple negative cancers was 18.1% among true interval cancers, 14.5% among minimal signs, and 9.6% among missed cancers.

**Table 2:** Histopathologic and clinicopathologic tumour characteristics of true, minimal signs, and missed screen-detected and interval breast cancers (proportions with 95% confidence intervals, CIs, unless otherwise specified)

Tumour characteristic	Screen-detected breast cancers						Interval breast cancers					
	True		Minimal signs		Missed		True		Minimal signs		Missed	
	*n* = 457		*n* = 336		*n* = 229		*n* = 310		*n* = 254		*n* = 224	
	*n*	% (95% CI)	*n*	% (95% CI)	*n*	% (95% CI)	*n*	% (95% CI)	*n*	% (95% CI)	*n*	% (95% CI)
Type												
Invasive NST^a^	408	89.9 (86.7, 92.3)	280	84.1 (79.8, 87.6)	198	86.8 (81.8, 90.6)	265	86.6 (82.3, 90.0)	212	83.8 (78.8, 87.8)	186	83.4 (78.0, 87.7)
Lobular	35	7.7 (5.6, 10.5)	37	11.1 (8.2, 14.9)	20	8.8 (5.8, 13.2)	35	11.4 (8.3, 15.5)	33	13.0 (9.4, 17.8)	26	11.7 (8.1, 16.5)
Other	11	2.4 (1.4, 4.3)	16	4.8 (3.0, 7.7)	10	4.4 (2.4, 7.9)	6	2.0 (0.9, 4.2)	8	3.2 (1.6, 6.1)	11	4.9 (2.8, 8.6)
Information not available	3	-	3	-	1	-	4	-	1	-	1	-
Tumour diameter (mm)												
Median (95% CI)	457	12 (11, 13)	336	13 (12, 14)	229	13 (12, 14)	310	19 (18, 20)	254	20 (18, 22)	224	20 (18, 22)
Histologic grade												
1	103	22.9 (19.2, 27.0)	111	33.0 (28.2, 38.2)	73	32.3 (26.5, 38.6)	26	8.5 (5.9, 12.2)	43	17.3 (13.1, 22.5)	30	13.8 (9.9, 19.0)
2	212	47.1 (42.5, 51.7)	175	52.1 (46.7, 57.4)	122	54.0 (47.5, 60.4)	137	44.8 (39.3, 50.4)	116	46.6 (40.5, 52.8)	109	50.2 (43.6, 56.8)
3	135	30.0 (25.9, 34.4)	50	14.9 (11.5, 19.1)	31	13.7 (9.8, 18.8)	143	46.7 (41.2, 52.3)	90	36.1 (30.4, 42.3)	78	35.9 (29.9, 42.5)
Information not available	7	-	0	-	3	-	4	-	5	-	7	-
Lymph node status												
Negative	352	78.0 (74.0, 81.6)	264	80.7 (76.1, 84.6)	181	80.4 (74.8, 85.1)	164	53.9 (48.3, 59.5)	153	61.0 (54.8, 66.8)	126	57.5 (50.9, 63.9)
Information not available	6	-	9	-	4	-	6	-	3	-	5	-
Subtype												
Luminal A-like	121	29.0 (24.9, 33.5)	114	37.4 (32.1, 42.9)	76	36.4 (30.1, 43.1)	31	11.0 (7.9, 15.2)	45	19.7 (15.1, 25.4)	52	25.0 (19.6, 31.3)
Luminal B-like	239	57.3 (52.5, 62.0)	174	57.0 (51.4, 62.5)	123	58.9 (52.1, 65.3)	175	62.3 (56.5, 67.7)	141	61.8 (55.4, 67.9)	118	56.7 (49.9, 63.3)
HER2+^b^ (non-luminal)	16	3.8 (2.4, 6.1)	10	3.3 (1.8, 5.9)	4	1.9 (0.7, 4.8)	24	8.5 (5.8, 12.4)	9	3.9 (2.1, 7.3)	18	8.7 (5.5, 13.3)
Triple negative (ductal)	41	9.8 (7.3, 13.1)	7	2.3 (1.1, 4.7)	6	2.9 (1.3, 6.1)	51	18.1 (14.1, 23.1)	33	14.5 (10.5, 19.6)	20	9.6 (6.3, 14.4)
Information not available	40	-	31	-	20	-	29	-	26	-	16	-

Note: 95% confidence intervals were calculated for proportions the Wilson score interval, and for medians using quantile regression with standard errors based on 100 bootstrap replications

^a^*NST* no special type

^b^Human epidermal growth factor receptor 2 positive

### Survival

Median follow-up was 5.4 years (range 0.2–12.8) for women with screen-detected cancer; 43 (4.2%) died from any cause and 10 (1.0%) died from breast cancer. Among women with interval cancer, median follow-up was 5.6 years (range 0.3–14.8); 81 (10.3%) died from any cause and 39 (4.9%) died from breast cancer.

**Fig. 2:**
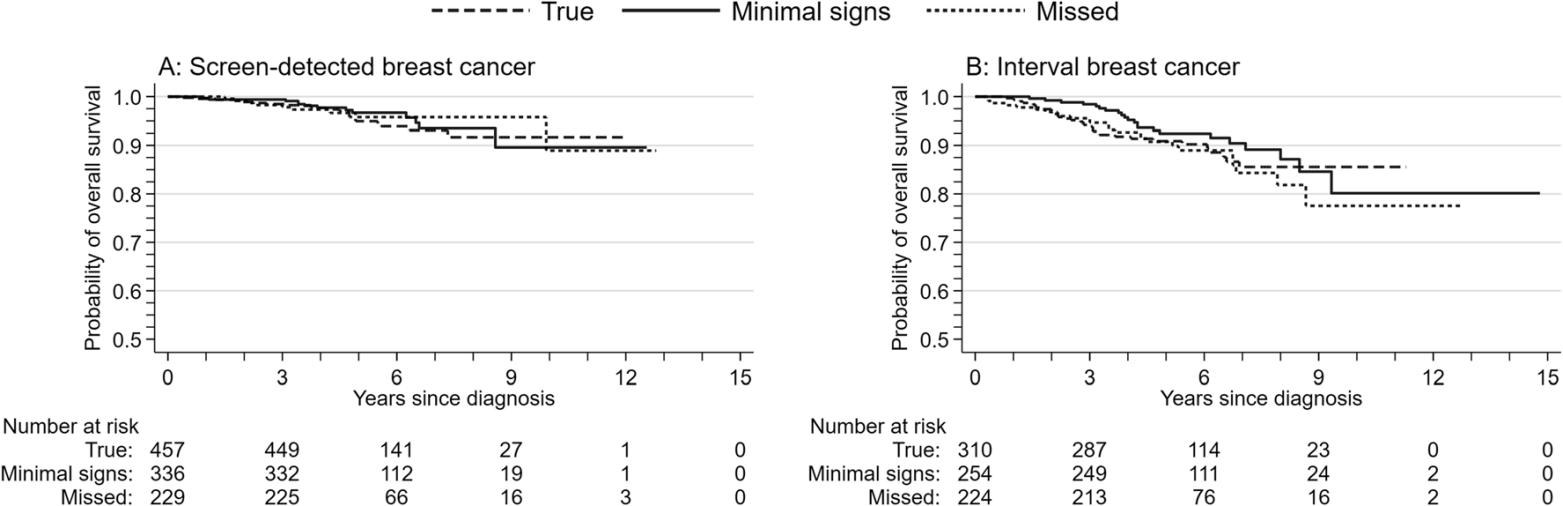
Kaplan-Meier estimates of overall survival for true, minimal signs, and missed (**a**) screen-detected and (**b**) interval breast cancers

**Fig. 3:**
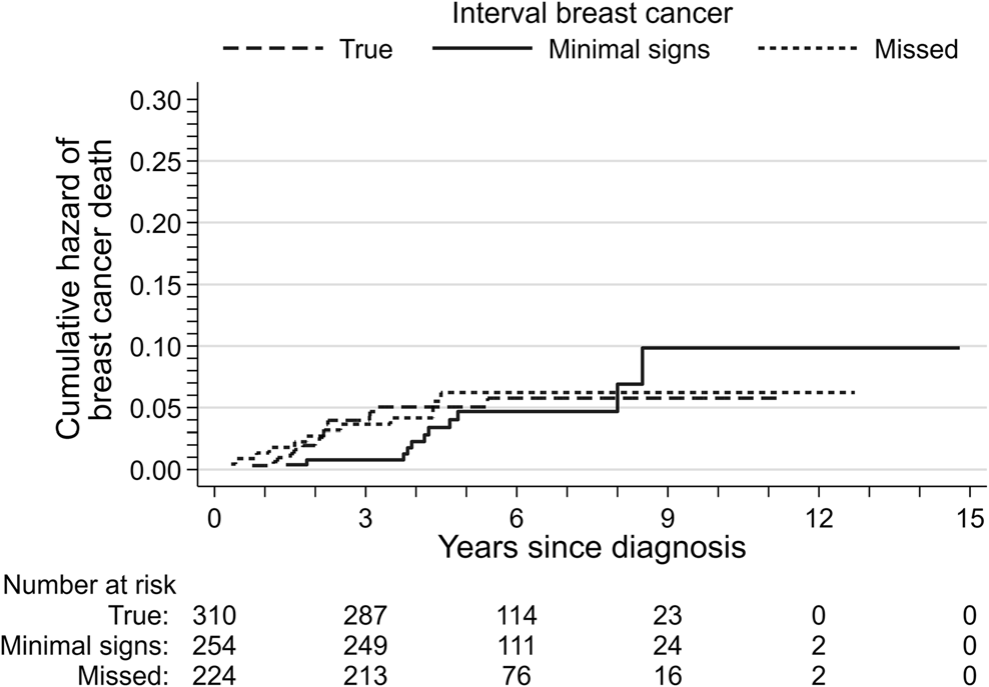
Nelson-Aalen cumulative hazard estimates of breast cancer death for true, minimal signs, and missed interval breast cancers

The Kaplan-Meier estimates for overall survival did not differ between true, minimal signs, and missed cancers, whether they were screen-detected (*p* = 0.82, Fig. 2a) or interval cancers (*p* = 0.43, Fig. 2b). We did not examine the Nelson-Aalen cumulative hazard for the risk of screen-detected breast cancer death because of the small number of deaths (5, 3, and 2 deaths among true, minimal signs, and missed cancers). The Nelson-Aalen cumulative hazard estimates of the risk of interval breast cancer death did not differ for true (16 deaths), minimal signs (11 deaths), or missed cancers (12 deaths; *p* = 0.80, Fig. 3).

The distribution of the imputed variables was comparable with that observed in complete case data (Table S2), and the results for complete case and multiple imputation analyses were similar (Table 3). We report the multiple imputation results here. In the multivariable Cox regression (Table 3), risk of death from any cause did not differ between minimal signs and true screen-detected cancers (HR = 1.04, 95% CI (0.51, 2.13)), or between missed and true screen-detected cancers (HR = 1.10, 95% CI (0.49, 2.46)). Similarly, the average risk of death from any cause during the entire follow-up period did not differ between minimal signs and true interval cancers (HR = 0.80, 95% CI (0.46, 1.37)), or missed and true interval cancers (HR = 1.31, 95% CI (0.77, 2.23)). Due to lack of proportional hazards, follow-up time was split for interval cancers. The risk of death among women with minimal signs interval cancers was lower than for women with true interval cancers during the first 3 years after diagnosis (HR = 0.29, 95% CI (0.10, 0.86)), but did not differ after the first 3 years (HR = 1.40, 95% CI (0.70, 2.80)). The risk of death from any cause did not differ for missed and true interval cancers before or after 3 years of follow-up.

**Table 3:** Hazard ratios (HRs) with 95% confidence intervals (CIs) for death due to any cause among women diagnosed with screen-detected and interval breast cancers

	Complete case						Multiple imputation^b^			
	No. of subjects	No. of deaths	Age adjusted		Multivariable^a^		Age adjusted		Multivariable^c^	
			HR	95% CI	HR	95% CI	HR	95% CI	HR	95% CI
Screen-detected breast cancers	921	35								
True	410	16	1.00	-	1.00	-	1.00	-	1.00	-
Minimal signs	305	11	0.93	(0.43, 2.00)	1.05	(0.48, 2.31)	0.87	(0.43, 1.73)	1.04	(0.51, 2.13)
Missed	206	8	1.12	(0.48, 2.63)	1.28	(0.53, 3.07)	0.90	(0.41, 1.97)	1.10	(0.49, 2.46)
Interval breast cancers, overall	702	73								
True	278	32	1.00	-	1.00	-	1.00	-	1.00	-
Minimal signs	223	18	0.69	(0.39, 1.23)	0.76	(0.42, 1.36)	0.77	(0.45, 1.32)	0.80	(0.46, 1.37)
Missed	201	23	1.02	(0.59, 1.76)	1.23	(0.71, 2.14)	1.13	(0.67, 1.90)	1.31	(0.77, 2.23)
Interval breast cancers, first 3 years^d^	702	29								
True	278	18	1.00	-	1.00	-	1.00	-	1.00	-
Minimal signs	223	3	0.21	(0.06, 0.70)	0.23	(0.07, 0.78)	0.27	(0.09, 0.79)	0.29	(0.10, 0.86)
Missed	201	8	0.65	(0.28, 1.51)	0.83	(0.35, 1.96)	0.83	(0.38, 1.81)	1.01	(0.45, 2.25)
Interval breast cancers, after 3 years^d^	673	44								
True	260	14	1.00	-	1.00	-	1.00	-	1.00	-
Minimal signs	220	15	1.31	(0.63, 2.73)	1.46	(0.70, 3.05)	1.34	(0.67, 2.66)	1.40	(0.70, 2.80)
Missed	193	15	1.51	(0.72, 3.17)	1.76	(0.83, 3.72)	1.50	(0.74, 3.07)	1.67	(0.81, 3.44)

^a^Adjusted for age at diagnosis, histopathologic tumour diameter and grade, and subtype

^b^Multiple imputation analyses conducted using chained equations and 40 generated data sets using 1022 screen-detected cancers (43 deaths) and 788 interval breast cancers (81 deaths)

^c^Model for screen-detected cancer adjusted for age at diagnosis, histopathologic tumour diameter and grade, and subtype. Models for interval cancer adjusted for age at diagnosis, histopathologic tumour diameter, and histopathologic grade, and stratified by subtype

^d^Follow-up time was split at 3 years due to lack of proportional hazards, the multiple imputation analyses included 788 interval cancers (32 deaths) in the model for the first 3 years, and 749 interval cancers (49 deaths) in the model for after 3 years

Results from the sensitivity analysis did not change our main conclusions (Table S3). This analysis included women without tumour diameter information, and did not use tumour diameter as a covariate in the imputation or Cox regression models.

## Discussion

We observed that true screen-detected and interval cancers had less favourable histopathologic characteristics than minimal signs and missed cancers in BreastScreen Norway. However, we did not observe any differences in the overall survival between these groups 3 years after diagnosis within each mode of detection after adjusting for age at diagnosis, tumour diameter, grade, and subtype. Our study included only women whose histopathologic tumour diameter was available (97.7% of screen-detected and 92.7% of interval cancers). For these women, there may not be substantial inherent prognostic differences associated with the classification of a true versus a missed or minimal signs cancer within a given mode of diagnosis (screen-detected or interval cancer). Our study is, as far as we know, the first to report overall survival among true, minimal signs, and missed screen-detected and interval breast cancers detected exclusively with digital mammography.

Missed screen-detected breast cancers could represent underdiagnosis if they have aggressive tumour characteristics at diagnosis, or overdiagnosis if they are indolent tumours. In our study, missed screen-detected cancers were often invasive NST, Luminal B-like, without lymph node involvement. Assuming the majority of these cancers never displayed any clinical symptoms, we conject that women with missed screen-detected cancer were not underdiagnosed and would not have benefitted from an earlier diagnosis at the prior screen. On the other hand, if these missed screen-detected cancers were overdiagnosed, they would have a longer lead time and higher survival than true screen-detected cancers. We did not observe higher overall survival for missed screen-detected cancers. Breast cancer–specific survival outcomes will provide more information about potential overdiagnosis, but longer follow-up is needed to obtain sufficient statistical power.

The literature suggests that true interval cancers are more likely to be smaller [17, 18, 20–22] and histologic grade 3 [17–19, 21, 22] than missed interval cancers. Our results confirmed that true interval cancers are more likely to be grade 3, but found no more than a 1 mm difference in the median histopathologic tumour diameter of true, minimal signs, and missed cancers. In our study and others, such findings about tumour diameter only apply to women for whom this information was available. Contemporary use of neoadjuvant therapy may have narrowed the observed range of tumour diameters in our study compared with older studies that took place when neoadjuvant therapy was less common. Moreover, our results may differ from those in previous studies that calculated a mean [17, 21], which is sensitive to the skewed distribution of tumour diameter, or that used a categorical variable [18, 21, 22], which may be misclassified at commonly used cut-points like 10 and 20 mm [33–35].

The tumour histopathology for true interval cancers indicated these were less favourable than minimal signs or missed cancers. However, we did not observe any differences in the overall survival or risk of death from breast cancer between true and minimal signs cancers 3 or more years following diagnosis, or between true and missed cancers during the entire follow-up period. We observed that minimal signs cancers were associated with a lower risk of death from any cause than true cancers during the first 3 years following diagnosis, even after adjustment for tumour histopathology. Our study is the first to report this finding and further studies are needed to confirm this result. Effective treatment options for advanced cancers may partially explain why the longer-term survival was similar for all classifications, despite differences in tumour histopathology. Our study did not include information about treatment regimens or long-term side effects which have the potential to highlight quality of life differences. Data completeness for oncological treatment is increasing at the national quality registry for breast cancer, and improved reporting of this information to the CRN may facilitate this type of analysis in the future [36]. In the absence of treatment data, longer follow-up may help us understand whether this survival profile for true, minimal signs, and missed interval cancers persists over a longer period, or whether any potential “treatment effect” is temporary.

Reviews are usually performed in a study setting with an artificially high volume of cancers, and radiologists are aware that they are being studied. This was also the case in our study and may limit the external generalizability of our results. Moreover, the distribution of true and missed cancers is sensitive to the review design used: higher proportions of missed interval cancers are associated with informed [13–15], and non-mixed reviews where cancer cases are not mixed in with negative screening examinations [11]. Lower proportions of missed interval cancers are associated with consensus-based reviews [15]. In our study, a panel of internal and external radiologists conducted a consensus-based informed review with one radiologist (T.H.) present during all classification activities to ensure methodological consistency. The panel had access to information about tumour localisation and features from diagnostic imaging, which may have led to a higher proportion of missed cancers in our study compared with studies with alternative review designs [21–23, 37].

Our study did not provide information about whether missed interval cancers would have a favourable prognosis had they been detected earlier as screen-detected breast cancers. Moreover, the statistical power was limited by the number of cases that the radiologists were able to review because review studies are resource intensive. Deep learning algorithms have the potential to interpret digital mammograms with a sensitivity and specificity comparable with radiologists, and there is now a focus on the potential for such algorithms to triage or identify true-negative screens so expert radiologists can focus on more challenging cases [38]. True interval cancers are the most frequent classification assigned in review studies [39] and using this technology to classify prior screening mammograms could substantially reduce the review workload for radiologists and facilitate larger studies than those conducted to date.

Unavailable histopathology data further limited the amount of information available for analysis—this is a common limitation of regression-based analyses because statistical software typically handles missing data by deleting the associated case [40]. We used multiple imputation to overcome the challenge of missing data and observed similar results from complete case and multiple imputation analyses. We could not impute tumour diameter information because it was not missing completely at random; therefore, we excluded women without this information from our sample. By excluding women whose histopathologic tumour diameter was not recorded at the CRN, we likely excluded women who received neoadjuvant therapy to downstage their tumour prior to surgery, thereby excluding women with the most aggressive tumours, particularly for interval cancers. Indeed, women without tumour diameter information in our study were more likely to have died during the follow-up period than women for whom this information was available. We performed a sensitivity analysis in which we included women without tumour diameter information, and did not use tumour diameter as a covariate in the imputation or Cox regression models. The results of this analysis did not change our main conclusions. However, we caution against generalising the results of our study to women who undergo neoadjuvant therapy.

The overall Cox regression model for interval cancers provided an estimate of the average risk of death over time. The proportional hazards assumption was violated in that model, which indicated that the risk of death in our sample was not constant over time. The models with split follow-up time indicated how that risk changed over time, but included fewer cases and had less statistical power than the overall model. Nonetheless, our study is one of the largest to evaluate the overall survival associated with true, missed, and minimal signs cancers in population-based screening. This is an important methodological strength of our work, as the adjusted complete case analyses omitted roughly 10% the available observations. Another strength of our study is that our sample included only cases detected with standard digital mammography, which is the current standard of care.

## Conclusion

We did not observe any differences in the longer-term overall survival between women classified as having true, minimal signs or missed screen-detected or interval cancers. However, the number of cases reviewed and follow-up time limited the precision of our estimates. In the future, deep learning algorithms may increase the number of prior screening mammograms that can be reviewed and thereby facilitate the analysis of breast cancer–specific survival associated with these classifications. This could provide additional information about the potential for “under” or “over” diagnosed breast cancer.

## Electronic supplementary material

ESM 1 (DOCX 25.8 kb)

## Abbreviations

CI: Confidence interval
CRN: Cancer Registry of Norway
DCIS: Ductal carcinoma in situ
ER: Estrogen receptor
HER2: Human epidermal growth factor receptor 2
PACS: Picture archiving and communication system
PR: Progesterone receptor
