# Supplementary material for: Survival among women diagnosed with screen-detected or interval breast cancer classified as true, minimal signs, or missed through an informed radiological review
Source: Eur Radiol. 2020 Nov 12;31(5):2677–86. doi: 10.1007/s00330-020-07340-4 (PMC8043922; doi:10.1007/s00330-020-07340-4)
Supplement: Supplementary file 1 — (DOCX 25.8 kb) [file 330_2020_7340_MOESM1_ESM.docx]

**Supplemental material**

**Table S1:** Definitions of tumour characteristics used in this study

| **Variable** | **Definition** |
| --- | --- |
| Histopathologic tumour diameter | Measured to the nearest mm using a transparent ruler and described the distance between the outermost boundaries of an invasive lesion from microscopic slides [1].  If a measurement could not be obtained using the microscopic slide, one was taken from the macroscopic examination (formalin fixed specimen). This measurement was taken either from a single tissue slice, or estimated across all tissue slices containing microscopically verified invasive tumour tissue [2]. |
| Lymph node status | Positive if micrometastases or metastases were detected in one or more regional or axillary lymph nodes. We prioritized histopathologic results from the diagnostic biopsy and used histopathologic results from the surgical specimen if the former were not available. |
| Estrogen receptor (ER) status | Positive if the sample displayed ≥10% reactivity, and negative otherwise. We prioritized histopathologic samples from the diagnostic biopsy and used histopathologic results from the surgical specimen if the former were not available. |
| Progesterone receptor (PR) status | Positive if the sample displayed ≥10% reactivity, and negative otherwise. We prioritized histopathologic samples from the diagnostic biopsy and used histopathologic results from the surgical specimen if the former were not available. |
| HER2^a^ positivity | If in situ hybridization (ISH) was performed, a borderline or amplified result was considered positive. If ISH was not performed, positivity was determined using immunohistochemistry (IHC). |
| Ki-67 expression | Percentage of Ki-67 positive cells from a sample of 500 tumour cells from the surgical specimen [1] |
| Subtype | Clinico-pathologic surrogate definition of intrinsic subtype based on the St Gallen consensus [3]  Luminal A:  ER and PR positive, HER2 negative, and Ki67 proliferation < 20%  Luminal B:  ER positive, PR negative and HER2 negative; or  ER positive, HER2 negative and Ki67 proliferation ≥ 20%; or  ER positive and HER2 positive  HER2 positive (non-luminal):  ER and PR negative, and HER2 positive  Triple negative (ductal):  ER, PR, and HER2 negative |

^a^ Human epidermal growth factor receptor 2

[1] Brystkreft – handlingsprogram [Internet]. Oslo: ​Norwegian Directorate of Health; 2019. [updated 2019/01/17; cited 2019/04/05]. Available from: <https://helsedirektoratet.no/retningslinjer/nasjonalt-handlingsprogram-med-retningslinjer-for-diagnostikk-behandling-og-oppfolging-av-pasienter-med-brystkreft>

[2] Badve SS, Beitsch PD, Bose S, et al. Part XI, Breast In: Amin MB, Edge S, Greene F, et al. editors. AJCC Cancer Staging Manual. 8th ed. Heidelberg, (Germany): Springer International Publishing; 2017.

[3] Goldhirsch A, Winer EP, Coates AS, Gelber RD, Piccart-Gebhart M, Thurlimann B, et al. Personalizing the treatment of women with early breast cancer: highlights of the St Gallen International Expert Consensus on the Primary Therapy of Early Breast Cancer 2013. Ann Oncol. 2013;24(9):2206-23.

| **Table S2**: Distribution of histopathologic and clinico-pathologic tumour characteristics (proportions with 95% confidence intervals, CIs^a^, unless otherwise specified) for true, minimal signs, and missed screen-detected and interval breast cancers based on 40 imputations | | | | | | |
| --- | --- | --- | --- | --- | --- | --- |
|  | **Screen-detected breast cancers** | | | **Interval breast cancers** | | |
| **Tumour characteristic** | **True** | **Minimal signs** | **Missed** | **True** | **Minimal signs** | **Missed** |
|  | **n = 457** | **n = 336** | **n = 229** | **n = 310** | **n = 254** | **n = 224** |
| **Histologic grade** |  |  |  |  |  |  |
| 1 | 23.1 (19.2, 27.0) | 33.0 (28.0, 38.1)^c^ | 32.5 (26.4, 38.6) | 8.5 (5.4, 11.7) | 17.2 (12.5, 21.8) | 13.8 (9.2, 18.4) |
| 2 | 47.2 (42.6, 51.8) | 52.1 (46.7, 57.4)^c^ | 53.9 (47.4, 60.4) | 44.8 (39.2, 50.4) | 46.3 (40.1, 52.5) | 50.1 (43.4, 56.7) |
| 3 | 29.7 (25.5, 33.9) | 14.9 (11.1, 18.7)^c^ | 13.6 (9.2, 18.1) | 46.6 (41.0, 52.2) | 36.6 (30.6, 42.5) | 36.1 (29.8, 42.5) |
| **Lymph node status** |  |  |  |  |  |  |
| Negative | 78.1 (74.3, 81.9) | 80.8 (76.6, 85.1) | 80.5 (75.3, 85.7) | 53.8 (48.1, 59.4) | 61.0 (55.0, 67.0) | 57.6 (51.1, 64.2) |
| **Subtype** |  |  |  |  |  |  |
| Luminal A-like | 34.3 (29.6, 39.0) | 43.1 (37.3, 48.9) | 43.9 (36.9, 50.8) | 17.6 (12.8, 22.4) | 26.8 (20.4, 33.1) | 31.2 (24.5, 37.9) |
| Luminal B-like | 51.2 (46.3, 56.2) | 51.5 (45.6, 57.4) | 51.3 (44.3, 58.2) | 57.3 (51.3, 63.3) | 55.3 (48.2, 62.3) | 49.9 (42.7, 57.1) |
| HER2+^b^ (non-luminal) | 4.3 (2.3, 6.3) | 3.1 (1.2, 5.0) | 2.0 (0.1, 3.8) | 8.1 (5.0, 11.2) | 4.3 (1.7, 7.0) | 8.9 (5.1, 12.8) |
| Triple negative (ductal) | 10.2 (7.3, 13.0) | 2.3 (0.6, 3.9) | 2.9 (0.7, 5.1) | 17.1 (12.8, 21.3) | 13.6 (9.3, 17.9) | 10.0 (5.9, 14.0) |

*^a^ Calculated using the Wilson score interval
^b^ Human epidermal growth factor receptor 2 positive
c These proportions are the same as in Table 2 because there was no missing information about histopathologic grade among women with minimal signs screen-detected breast cancer*

**Table S3**: Hazard ratios (HRs) with 95% confidence intervals (CIs) for death due to any cause among women diagnosed with screen-detected and interval breast cancers included in the original analysis in the main text (Table 3) and in a sensitivity analysis that additionally included those that without tumour diameter information

|  | **Multivariable^a,b^ results from original analysis**  **(*excluding* women without tumour diameter information)** | | **Multivariable^a^ results from sensitivity analysis**  **(*including* women with without tumour diameter information)** | |
| --- | --- | --- | --- | --- |
|  | **HR** | **95% CI** | **HR** | **95% CI** |
| **Screen-detected breast cancers** |  |  |  |  |
| True | 1.00 | - | 1.00 | - |
| Minimal signs | 1.05 | (0.48, 2.31) | 1.09 | (0.55, 2.15) |
| Missed | 1.28 | (0.53, 3.07) | 1.15 | (0.54, 2.46) |
|  |  |  |  |  |
| **Interval breast cancers, overall** |  |  |  |  |
| True | 1.00 | - | 1.00 | - |
| Minimal signs | 0.76 | (0.42, 1.36) | 0.90 | (0.56, 1.45) |
| Missed | 1.23 | (0.71, 2.14) | 1.40 | (0.88, 2.22) |
|  |  |  |  |  |
| **Interval breast cancers, first 3 years** |  |  |  |  |
| True | 1.00 | - | 1.00 | - |
| Minimal signs | 0.23 | (0.07, 0.78) | 0.54 | (0.27, 1.10) |
| Missed | 0.83 | (0.35, 1.96) | 0.97 | (0.51, 1.86) |
|  |  |  |  |  |
| **Interval breast cancers, after 3 years** |  |  |  |  |
| True | 1.00 | - | 1.00 | - |
| Minimal signs | 1.46 | (0.70, 3.05) | 1.47 | (0.74, 2.91) |
| Missed | 1.76 | (0.83, 3.72) | 2.13 | (1.07, 4.24) |

^a^ Model for screen-detected cancer adjusted for age at diagnosis, and grade, and subtype. Models for interval cancer adjusted for age at diagnosis and histopathologic grade, and stratified by subtype

^b^ Models for screen-detected and interval cancer additionally adjusted for histopathologic tumour diameter
